# Congenital Hypothyroidism and Atherosclerosis: An Endocrine Model of Early-Life Cardiovascular Risk

**DOI:** 10.3390/jcdd13080369

**Published:** 2026-08-04

**Authors:** Maria Tzoraki, Leonidas H. Duntas

**Affiliations:** 1 Department of Endocrinology, Growth and Development, Children’s Hospital P. & A. Kyriakou, 11527 Athens, Greece; maria.tzoraki@gmail.com; 2 Unit of Endocrinology, Diabetes and Metabolism, Evgenideion Hospital, National and Kapodistrian University of Athens, 11528 Athens, Greece

**Keywords:** congenital hypothyroidism, atherosclerosis, endothelial dysfunction, intima–media thickness, arterial stiffness, thyroid-stimulating hormone, lipid metabolism, familial hypercholesterolemia, cardiovascular risk

## Abstract

Congenital hypothyroidism (CH) is a common endocrine disorder characterized by thyroid hormone deficiency and has dramatic consequences if diagnosis and treatment are delayed. Although neonatal screening (NNS) and early levothyroxine (L-T4) treatment have markedly improved neurodevelopmental outcomes, evidence suggests that cardiovascular alterations may persist into later life. Thyroid hormones regulate lipid metabolism, endothelial nitric oxide (NO) availability, myocardial relaxation, vascular tone and arterial wall homeostasis. Early clinical studies in patients with CH reported variable lipid and lipoprotein abnormalities, whereas more recent studies have demonstrated endothelial dysfunction, increased arterial stiffness, and elevated carotid intima–media thickness (IMT), even in the absence of classical cardiovascular risk factors. These alterations appear to be associated with cumulative exposure to thyroid-stimulating hormone (TSH) abnormalities rather than isolated biochemical measurements. These findings suggest that CH may affect cardiovascular biology through multiple pathways, including dyslipidemia, endothelial dysfunction, vascular remodeling, and cumulative exposure to thyroid-stimulating hormone abnormalities. This review summarizes the available evidence linking congenital hypothyroidism to early vascular and atherosclerotic changes and discusses its conceptual relationship to familial hypercholesterolemia as another model of early-life cumulative cardiovascular risk.

## 1. Introduction

Congenital hypothyroidism (CH) is an endocrine disorder characterized by thyroid hormone deficiency from birth, during a developmental period when thyroid hormones are essential not only for growth and maturation but also for metabolic regulation, cardiac function, and vascular homeostasis. Untreated CH is known as cretinism [1,2]. Neonatal screening (NNS) and early levothyroxine (L-T4) therapy have substantially improved the prognosis of CH. Increasing attention is now focused on long-term outcomes beyond neurodevelopment, including metabolic and cardiovascular health [2,3].

The cardiovascular relevance of CH stems from the key role of thyroid hormones in lipid metabolism, endothelial function, and vascular homeostasis, which are central to the development of atherosclerosis. Thyroid hormones regulate myocardial contractility, heart rate, systemic vascular resistance, endothelial function, and lipid metabolism [4,5]. In adults, overt and subclinical thyroid dysfunction have been associated with dyslipidemia, endothelial dysfunction, arterial stiffness, and increased cardiovascular risk [4,5,6]. These observations raise the question of whether thyroid hormone deficiency beginning in fetal or neonatal life may also influence cardiovascular development and early vascular health in patients with CH. A useful framework for interpreting these findings is the concept of cumulative exposure. In familial hypercholesterolemia (FH), lifelong exposure to elevated low-density lipoprotein cholesterol (LDL-C) from childhood is a well-established driver of premature atherosclerosis [7,8]. On the other hand, CH is not a classical lipid disorder and its cardiovascular profile differs from FH. However, it may offer a complementary model that accounts for early-life endocrine disturbances, variable lipid alterations, and long-term thyroid-stimulating hormone (TSH) fluctuations in relation to vascular changes.

Although randomized controlled studies (RCTs) are lacking and the existing data are scarce, this review aims to examine the available evidence linking CH to early cardiovascular and vascular alterations, with particular emphasis on lipid metabolism, endothelial function, vascular remodeling, and cumulative hormonal exposure.

## 2. Materials and Methods

This manuscript was designed as a narrative review. Relevant literature was identified through PubMed/MEDLINE searches and manual screening of reference lists, focusing on congenital hypothyroidism, thyroid hormone deficiency, lipid metabolism, endothelial dysfunction, arterial stiffness, intima–media thickness, flow-mediated dilation, and early markers of atherosclerosis. Additional mechanistic and guideline-based references were included when they provided context on thyroid hormones, vascular biology, apolipoprotein B-containing lipoproteins, or familial hypercholesterolemia as a model of cumulative cardiovascular risk. Priority was given to original clinical studies involving neonates, children, adolescents, and young adults with congenital hypothyroidism, together with consensus documents and reviews relevant to thyroid disease, lipid metabolism, and cardiovascular prevention. Because the purpose of the article was to provide a critical narrative synthesis rather than a systematic review or meta-analysis, no formal PRISMA-based selection process, risk-of-bias assessment, or quantitative synthesis was performed. Generative artificial intelligence was used only for language editing and paraphrasing during manuscript preparation; all scientific content and references were reviewed and approved by the authors.

## 3. Thyroid Hormones, Lipid Metabolism, and Vascular Function

Thyroid hormones regulate lipid metabolism, primarily through their effects on hepatic lipid handling and lipoprotein turnover [9]. Triiodothyronine (T3) enhances the expression of hepatic LDL receptors, thereby promoting the clearance of circulating LDL particles, and also modulates cholesterol synthesis, bile acid metabolism, and the activity of enzymes involved in lipoprotein remodeling, such as hepatic lipase and lipoprotein lipase [5,10]. When thyroid hormone activity is decreased, these processes are impaired, leading to reduced LDL clearance and accumulation of atherogenic apolipoprotein B (apo B)-containing lipoproteins [11]. Consistent with this, hypothyroidism is a well-recognized secondary cause of hypercholesterolemia and is routinely considered during the diagnostic evaluation of dyslipidemia, including in patients with suspected familial hypercholesterolemia [7,8].

Beyond lipids, thyroid hormones also influence vascular homeostasis. They modulate endothelial nitric oxide (NO) availability, vascular smooth muscle relaxation, and systemic vascular resistance [4]. In hypothyroid states, reduced NO bioavailability and increased vascular tone contribute to endothelial dysfunction and arterial stiffness, both of which represent early functional changes in atherogenesis [12].

This dual metabolic and vascular role provides the biological basis for examining lipid and vascular findings specifically in CH.

## 4. Lipid and Lipoprotein Findings in CH

Early studies of lipid metabolism in congenital hypothyroidism were relatively small and heterogeneous, but they remain important because they provide rare data on lipid and lipoprotein profiles during infancy and childhood.

In a small cohort of infants, Asami et al. (1995) [13] evaluated 8 infants with CH and 10 healthy controls at approximately 1 month of age and again after L-T4 treatment. Before treatment, infants with CH had significantly higher total cholesterol (TC), low-density lipoprotein cholesterol (LDL-C), and high-density lipoprotein cholesterol (HDL-C) compared with controls. After L-T4 therapy, TC and HDL-C decreased toward control values, whereas LDL-C showed a variable response and did not normalize consistently. The atherogenic index was also higher in CH and increased further after treatment [13].

The same research group later investigated whether CH affects not only lipid concentrations but also lipoprotein composition. In a study by Asami et al. (1999a) [14], 17 infants with CH were assessed before and after L-T4 therapy, with measurement of apolipoproteins (apo), including apo A-I, apo A-II, apo B, apo C-II, apo C-III, and apo E. After treatment, HDL, apo A-I, apo C-III, and apo E decreased, while apo B increased. TC and LDL-C did not change significantly. The increase in apo B is particularly relevant, as apo B-containing particles are central to atherogenic risk [14].

However, the lipid profile in CH is not uniform across studies. Ciomartan et al. (1999) [15] studied a larger cohort of 75 untreated infants with CH. Mean lipid values were generally within normal ranges, but a subset of patients had elevated TC and LDL-C. Specifically, 26.6% had elevated total cholesterol, and 13.3% had elevated LDL-C. Lipid parameters did not correlate significantly with TSH or free thyroxine (FT4), while T3 showed an inverse association with TC and LDL-C [15].

Further mechanistic information comes from the study of Asami et al. (1999b) [16] on lipoprotein fractions in older children with CH. This cohort included 19 children aged 9 months to 12 years, compared with 13 children without thyroid disease. The study did not show a significant difference in the overall proportion of intermediate-density lipoprotein (IDL) between CH patients and controls. However, FT4 levels were inversely correlated with IDL, and IDL was positively associated with total cholesterol, apo B, and apo C-II. These observations indicate that thyroid hormone status may influence lipoprotein remodeling, particularly IDL metabolism, even when group differences in standard lipid fractions are not apparent [16].

Overall, these early studies suggest that lipid findings in CH are heterogeneous. They do not establish a uniform dyslipidemic phenotype, but they indicate that thyroid hormone deficiency may influence lipid concentrations, apolipoprotein composition, and lipoprotein remodeling in early life. Beyond metabolic alterations, subsequent studies have suggested that CH may also affect myocardial function, endothelial activation, and vascular structure from early life.

## 5. Cardiovascular and Vascular Phenotype in CH: From Early Functional Changes to Subclinical Atherosclerosis

Early evidence of cardiovascular involvement in CH is detectable at the myocardial level. Arslan et al. demonstrated that neonates with CH exhibit impaired diastolic function, as reflected by a reduced early-to-late ventricular filling velocity ratio (E/A ratio) and an increased myocardial performance index, while systolic function remains preserved. These abnormalities improved following L-T4 replacement, suggesting partial reversibility [17].

Vascular alterations appear to occur in parallel and provide a direct link to atherogenesis. Endothelial dysfunction, defined as impaired endothelium-dependent vasodilation and a pro-inflammatory endothelial phenotype, represents an early and pivotal step in atherosclerosis [18,19]. In neonates with CH, Hashemipour et al. [20] reported increased circulating levels of intercellular adhesion molecule-1 (ICAM-1) and vascular cell adhesion molecule-1 (VCAM-1), both key mediators of leukocyte adhesion to the endothelium. These molecules facilitate monocyte recruitment into the subendothelial space, a critical initiating event in fatty streak formation. Importantly, their levels decreased after L-T4 therapy, suggesting that endothelial activation is, at least partially, reversible [20].

Structural vascular changes are also detectable early. Akin et al. [21] demonstrated increased abdominal aortic intima–media thickness (IMT) in neonates with CH compared with controls. IMT is a well-established surrogate marker of early atherosclerosis, reflecting vascular wall remodeling. In this study, aortic IMT correlated positively with TSH and TC and negatively with fT4, indicating that both hormonal deficiency and lipid disturbances contribute to early vascular changes [21].

The persistence of these abnormalities into adulthood further supports the concept of a lifelong vascular phenotype. Salerno et al. [22] showed that young adults with CH, despite early diagnosis and long-term L-T4 treatment, exhibit impaired left ventricular diastolic function, reduced exercise capacity, and increased carotid IMT. Notably, these alterations were not related to thyroid hormone levels at the time of assessment but to the cumulative number of episodes of TSH dysregulation over the life course [22].

Consistent with this, Oliviero et al. [23] demonstrated impaired endothelial function in young adults with CH, as assessed by reduced flow-mediated dilation (FMD), a measure of NO-dependent vasodilation. These patients also exhibited reduced arterial distensibility, reflecting increased vascular stiffness that was independent of traditional cardiovascular risk factors such as body mass index, blood pressure, and lipid levels, and was instead strongly associated with mean TSH levels during puberty and episodes of undertreatment [23].

Recent data further support a dynamic interaction between thyroid hormones, lipid metabolism, and vascular structure. Soliman et al. [24] reported that children with hypothyroidism, including those with CH, exhibit increased TC, triglycerides, and LDL-C, together with increased aortic IMT. Following L-T4 therapy, both lipid parameters and aortic IMT decreased significantly, indicating that thyroid hormone replacement can partially reverse vascular remodeling. However, the persistence of abnormalities in some patients suggests that early exposure may have lasting effects [24].

**Figure 1:**
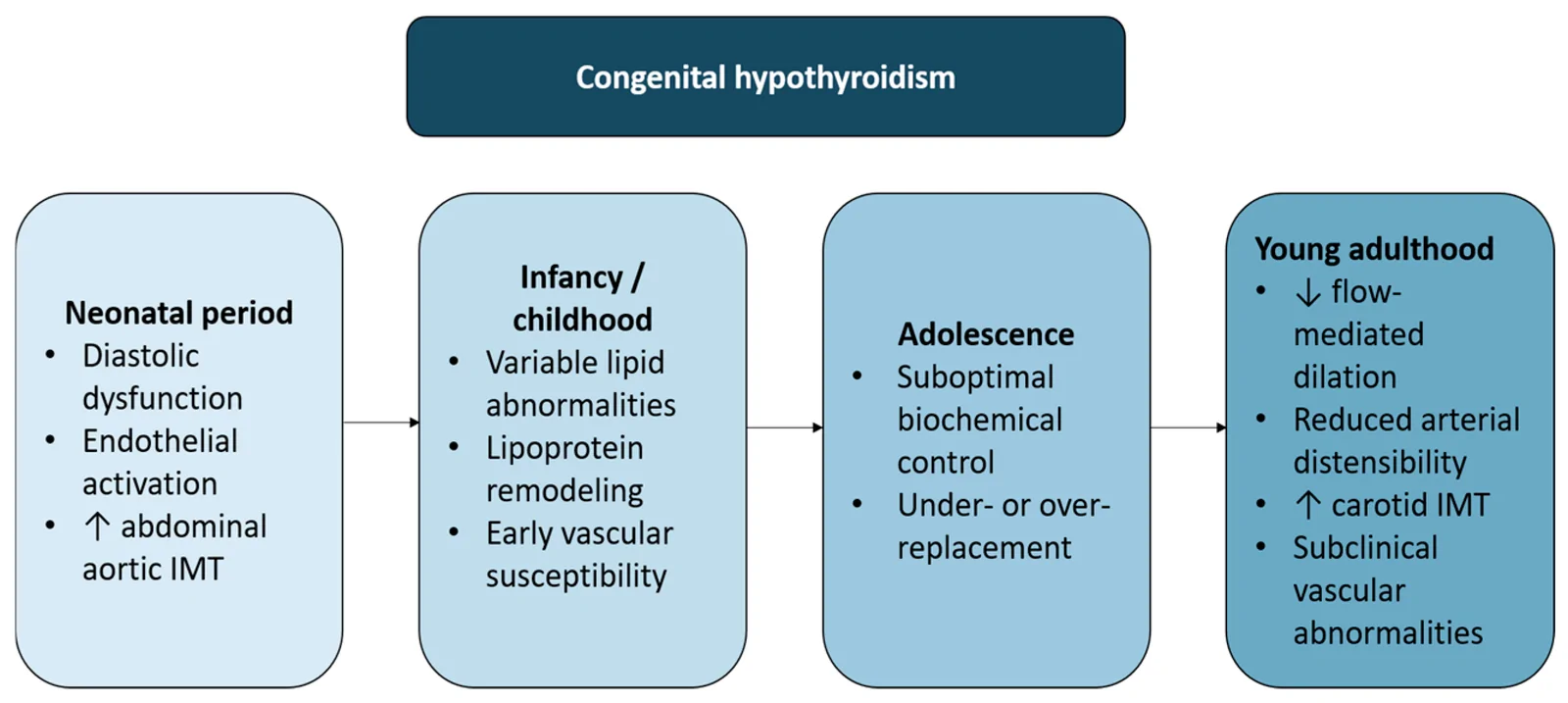
Proposed life-course evolution of vascular alterations in patients with congenital hypothyroidism despite early diagnosis and levothyroxine replacement. Reported abnormalities include early endothelial activation and increased abdominal aortic intima–media thickness during the neonatal period, lipid and lipoprotein alterations during childhood, treatment-related biochemical instability during adolescence, and persistent subclinical vascular abnormalities in young adulthood. The figure summarizes findings reported in the literature and does not imply a uniform progression in all patients. Abbreviations: IMT, intima–media thickness. Symbols: ↑ indicates an increase; ↓ indicates a decrease.

These findings can be integrated into a life course model in which CH is associated with early myocardial involvement, endothelial activation, vascular remodeling, and later subclinical cardiovascular alterations, as shown in Figure 1.

## 6. Mechanistic Pathways Linking CH to Vascular Injury

The available evidence suggests that vascular involvement in CH is unlikely to be explained by a single pathway. Rather, it appears to reflect the convergence of metabolic, endothelial, and hormonal mechanisms. Reduced thyroid hormone availability may impair NO-mediated vasodilation and increase vascular tone, while endothelial activation may promote leukocyte adhesion and low-grade vascular inflammation [18,19]. In parallel, variable abnormalities in LDL-C and apo B-containing particles may contribute to lipid retention within the vascular wall in susceptible patients [11]. Repeated episodes of elevated TSH or undertreatment may further amplify these processes over time, particularly during periods of rapid growth such as puberty [22,23].

**Figure 2:**
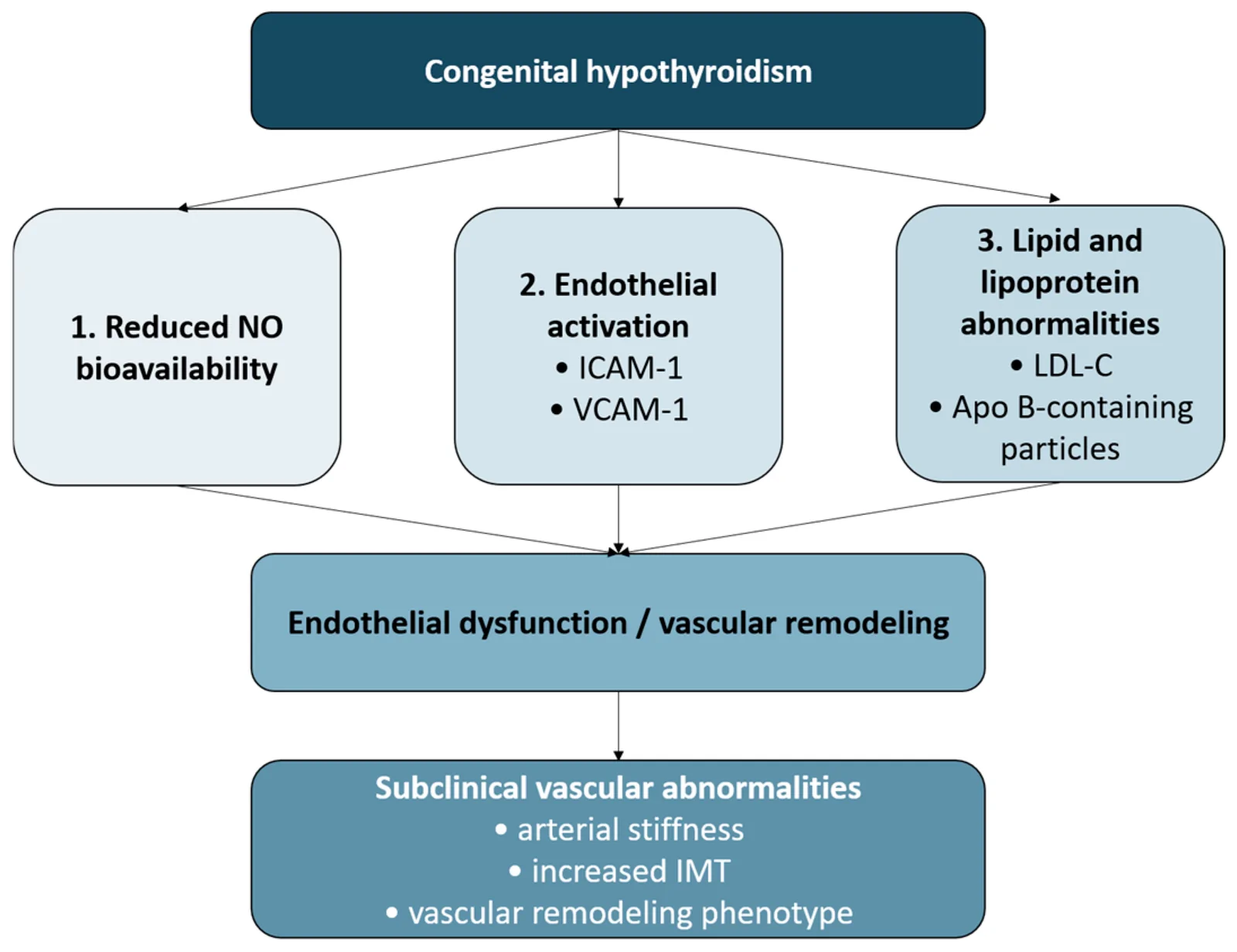
Proposed mechanisms linking congenital hypothyroidism to subclinical vascular abnormalities. Reduced nitric oxide bioavailability, endothelial activation, and lipid/lipoprotein abnormalities may contribute to endothelial dysfunction and vascular remodeling, ultimately leading to increased arterial stiffness and intima–media thickness. The proposed mechanisms are based on currently available evidence and remain hypothetical. Abbreviations: NO: nitric oxide; ICAM-1: Intercellular adhesion molecule-1; VCAM-1: Vascular cell adhesion molecule-1, LDL-C: low-density lipoprotein cholesterol; Apo B: apolipoprotein B; IMT: intima–media thickness.

This integrated model helps explain why vascular alterations in CH may be observed even when lipid abnormalities are inconsistent across studies and thyroid function appears normal at a single assessment [15,22,23]. The proposed mechanistic pathways linking CH with endothelial dysfunction and vascular injury are summarized in Figure 2.

## 7. Cumulative Exposure: Conceptual Parallels with FH

The comparison between CH and FH should remain conceptual. FH is a monogenic lipid disorder in which lifelong exposure to elevated LDL-C is the primary driver of premature atherosclerotic cardiovascular disease [7,8]. CH, in contrast, is an endocrine disorder with a more heterogeneous cardiovascular phenotype. Nevertheless, both conditions illustrate the importance of cumulative exposure from early life. In CH, the relevant burden is not limited to lipids but may include thyroid hormone deficiency, TSH variability, endothelial activation, and vascular remodeling [22,23].

## 8. Treatment Modalities in CH

There are two main types of CH: permanent (p-CH) and transient (t-CH). LT4 dosages above 4.7 µg/kg/day and below 1.8 µg/kg/day at age 1 year may help predict p-CH and t-CH, respectively [25]. CH has diverse genetic etiologies and variable clinical courses, ranging from transient neonatal hyperthyroidism to permanent thyroid hormone or thyrotropin deficiency [26]. Advances in molecular genetics have substantially expanded the catalog of genes implicated in CH [27]. The three LT4 formulations (tablet, drops, and solution) have been shown to be equally effective in normalizing and maintaining serum TSH and FT4 within reference ranges [28]. However, at 4 years of age, only the high-dose LT4 regimen of 10.1–15.0 µg/kg per day rapidly normalizes serum TSH concentrations and improves IQ, even in patients with severe CH at diagnosis [28]. Growth and bone age maturation are not affected by such a high dose. For transient CH, it is recommended that LT4 be discontinued after age 3 years, with further thyroid function and imaging evaluations [1,29].

In cases of secondary hypercholesterolemia due to underlying hypothyroidism, timely diagnosis and treatment with statins and/or ezetimibe, when adjusting hypothyroidism is insufficient, must be emphasized to prevent atherosclerosis [30].

Recently, the portfolio of lipid-lowering medications has been expanded to include PCSK9 inhibitors, which are small interfering RNA (siRNA) agents targeting proprotein convertase subtilisin/kexin type 9 (PCSK9) [31]. PCSK9 is an increasingly important target for cholesterol therapies. Treatment with PCSK9 inhibitors, such as inclisiran or evolokumab, may be advantageous for young patients and children with CH, FH, and lipoprotein (a) levels above 50 mg/L [31]. There is strong evidence supporting PCSK9 monoclonal antibodies for patients not eligible for other lipid-lowering drugs or unable to achieve lipid goals with traditional therapies. However, given the high heterogeneity across several estimates, funnel plot asymmetry, and the low number of studies, the findings should be interpreted cautiously pending cardiovascular outcomes [32].

## 9. Limitations of Current Evidence

Several limitations should be acknowledged. The number of studies directly examining congenital hypothyroidism and atherosclerosis is limited, and most cohorts are small. Early studies were conducted before current treatment protocols and involved heterogeneous patient populations. In addition, most available studies are observational or cross-sectional, limiting causal inference.

Most data rely on surrogate cardiovascular markers, including IMT, FMD, arterial distensibility, and exercise capacity, rather than hard cardiovascular outcomes such as myocardial infarction, stroke, or cardiovascular mortality. Lipid findings are also inconsistent across studies, suggesting that dyslipidemia alone cannot explain vascular changes in CH. Large prospective studies are therefore needed to determine whether these early abnormalities translate into clinically relevant cardiovascular risk later in life.

## 10. Future Directions

Extending NNS to many more countries than today is of utmost importance. Currently, 29.6% of children worldwide are screened for CH [33]. In recent decades, several countries have launched NNS programs for CH or conducted pilot studies, and the coverage of most existing NNS programs has increased. Nevertheless, approximately 70% of newborns worldwide still lack access to NNS programs, particularly in Asian and African countries [34].

Future research should establish large longitudinal cohorts of patients with CH to track their progress from diagnosis through adulthood. These studies are essential for determining whether early abnormalities in vascular function and structure, such as endothelial function and IMT, predict later cardiovascular outcomes. It is also critical to standardize vascular assessments in infants and children, as measurements can vary by age, body size, and imaging protocols. Abdominal aorta IMT measurement is a feasible method for early detection of atherosclerosis in CH and shows a significant decrease after treatment [24]. Achieving euthyroidism is associated with significant decreases in TC, triglycerides, and LDL-C, and an increase in HDL.

Additionally, future studies should examine TSH trajectories rather than relying solely on isolated thyroid function measurements. This is especially important during puberty and the transition from pediatric to adult care, when treatment adherence may become less consistent, and thyroid hormone requirements may change. Understanding whether optimized adherence to L-T4 and sustained euthyroidism can prevent or reverse endothelial dysfunction and vascular remodeling remains a crucial unanswered question.

Finally, mechanistic studies that incorporate biomarkers of NO bioavailability, oxidative stress, endothelial adhesion molecules, and apo B-containing particles may help clarify the biological pathways linking CH to early vascular injury.

## 11. Conclusions

CH should not be viewed solely as a neonatal endocrine disorder corrected by early L-T4 therapy. Although neurodevelopmental outcomes have improved substantially, accumulating evidence suggests an association with long-term cardiovascular and vascular alterations.

The available evidence supports a model in which early thyroid hormone deficiency, endothelial dysfunction, lipid disturbances, and cumulative TSH variability may interact to influence vascular health. In this context, CH may serve as a model of early-life endocrine dysregulation with potential implications for cardiovascular risk. Its comparison with FH should remain conceptual: both conditions demonstrate how early sustained biological exposure may shape long-term vascular outcomes, albeit through different primary mechanisms.

## Data Availability

No new data were created or analyzed in this study. Data sharing is not applicable to this article.

## Abbreviations

The following abbreviations are used in this manuscript:

CH	Congenital hypothyroidism
NNS	Neonatal screening
L-T4	Levothyroxine
FH	Familial hypercholesterolemia
LDL	Low-density lipoprotein
TSH	Thyroid-stimulating hormone
T3	Triiodothyronine
apo B	Apolipoprotein B
NO	Nitric oxide
TC	Total cholesterol
LDL-C	Low-density lipoprotein cholesterol
HDL-C	High-density lipoprotein cholesterol
apo	Apolipoproteins
apo A-I	Apolipoprotein A-I
apo A-II	Apolipoprotein A-II
apo C-II	Apolipoprotein C-II
apo C-III	Apolipoprotein C-III
apo E	Apolipoprotein E
FT4	Free thyroxine
IDL	Intermediate density lipoprotein
E/A ratio	Early-to-late ventricular filling velocity ratio
ICAM-1	Intercellular adhesion molecule-1
VCAM-1	Vascular cell adhesion molecule-1
IMT	Intima–media thickness
FMD	Flow-mediated dilation
p-CH	Permanent congenital hypothyroidism
t-CH	Transient congenital hypothyroidism
siRNA	Small interfering RNA
PCSK9	Proprotein convertase subtilisin/kexin type 9
